# The Effect of Fatty Acid Desaturase on Cardiovascular Lipid Biomarkers Depends on Circulating ω-3 and ω-6 Polyunsaturated Fatty Acids in the UK Biobank

**DOI:** 10.3390/nu17061089

**Published:** 2025-03-20

**Authors:** Carmen E. Arrington, Jason Westra, Nathan L. Tintle, Gregory C. Shearer

**Affiliations:** 1 Department of Nutritional Sciences, The Pennsylvania State University, 110 Chandlee Lab, University Park, PA 16802, USA; cearrington@ucdavis.edu; 2 The Fatty Acid Research Institute, Sioux Falls, SD 57106, USA; jason@faresinst.com (J.W.); nlt@faresinst.com (N.L.T.)

**Keywords:** polyunsaturated fatty acids, gene–nutrient interactions, cardiovascular disease, lipid biomarkers, fatty acid desaturase

## Abstract

Objectives: The objective of this study is to outline a framework for how fatty acids may be acting as mediators/suppressors and/or moderators of an individual’s genetic predisposition for cardiovascular lipid biomarkers. Methods: All UK Biobank participants with demographic and lifestyle variables, circulating cardiovascular lipids, and NMR-measured fatty acid data collected at the baseline visit (*N* = 229,859) were included in analyses. We fit four separate linear regression models, one for each of the following common measures of cardiovascular lipids: total cholesterol, HDL-c, LDL-c, and total triglycerides. Each model predicted cardiovascular lipids by an individual’s *FADS* (a well-known fatty acid desaturase gene complex) haplotype, with the addition of individual ω-3 (DHA, non-DHA, and total), ω-6 (LA, non-LA, and total), or SFA factors as additive (mediation/suppression) or using an interaction term (with *FADS*) (moderation). All models were adjusted for a wide range of demographic and medical history variables and evaluated against a Bonferroni-adjusted significance level (*p* < 8.9 × 10^−4^). Results: Across 56 models (four lipids × seven FAs × two conceptual models (mediation/suppression and moderation)), we found evidence of 19 moderation, 12 mediation, and 16 suppression effects of the *FADS*–lipid relationship. For example, adjusting for circulating DHA levels as a mediator, the association of the genotype with HDL-c substantially lessened for both minor genotypes reflecting >122% mediation of the association of *FADS* by DHA. Additionally, we found evidence that LDL-c is moderated, to some extent, by all fatty acid measures. Conclusions: This analysis demonstrates that an individual’s fatty acid profile can act as a mediator/suppressor or moderator of the association of the *FADS* genotype and various cardiovascular biomarkers. Future work is necessary to expand this cross-sectional examination to determine directionality and temporality of the mediation and moderation evidence presented. This research has been conducted using the UK Biobank Resource under Application Number 85092.

## 1. Introduction

The association between circulating fatty acid (FA) levels and cardiovascular disease (CVD) has received a wealth of attention; however, few studies have examined how genetic risk for CVD and risk factors may be impacted by polyunsaturated fatty acid (PUFA) profiles. FAs have a variety of characteristic functions including inter- and intracellular signaling [1,2] and membrane composition [3]. PUFAs are precursors for oxylipins, which are bioactive signaling molecules formed by enzymatic oxygenation [4]. Epidemiological [5,6,7] and large-scale randomized control studies have demonstrated the protective effects of omega-3 (ω-3) PUFAs, specifically eicosapentaenoic acid (EPA) and docosahexaenoic acid (DHA), in the development of CVD, including GISSI-P and JELIS [8]. However, ω-3 PUFA supplementation or pharmaceutical intervention trials have yielded mixed results due to variability in treatment time, dose, baseline PUFA status, and the inability to replicate the therapeutic effects of ω-3 PUFAs, such as fish oil, within short-duration trials [9,10,11,12]. These results may also be due to the population being studied and the potential for inclusion of “non-responders”, considered participants that do not benefit from a particular intervention, alongside classical “responder” participants who could benefit [13]. Elucidation of what determines why a participant is a “non-responder” is critical for further research into the therapeutic effects of PUFAs.

Emerging evidence suggests the potential differential benefits of FAs depends in part on genotype [14,15,16,17,18,19,20,21,22,23], with few studies specifically examining FA mediation of gene associations with adverse outcomes [24,25,26]. Recently, Chaaba et al. emphasized the extensive associations between FAs or genetic variants of proteins in FA metabolism with prevention and management of CVD, diabetes, and cancer; however, they did not investigate the potential inter-dependencies between FAs and genetic variants [27]. Here, we present an approach to explore circulating FAs as mediators and/or moderators of the FADS risk for lipid outcomes relevant to CVD. In completing the mediation analysis, we also examine the potential for suppression. Suppression occurs when the primary effect is not evident until the third factor is included [28]. To do this, we explore a particular set of genes, FAs, and lipid outcomes as a case study—both to shed light on these particular relationships but also to demonstrate the framework that can be used elsewhere. This case study focuses on the fatty acid desaturase (*FADS*) gene complex, a series of co-localized FA desaturating genes that encode the rate-limiting step in FA synthesis, which is the most well-characterized FA gene and explains the most variation in FA levels [18,22,23]. These genes can be summarized by a single, multi-gene spanning region that tends to exist as one of two primary haplotypes [29].

PUFA synthesis occurs via elongation and desaturation of essential dietary ω-3 and ω-6 PUFA LA and alpha-linolenic acid. The *FADS1* (chr11: 61,799,627–61,817,003) and *FADS2* (chr11: 61,816,203–61,867,354) genes are the rate-limiting steps in PUFA synthesis and have been previously characterized into an *FADS* gene complex, encompassing both the Δ5 desaturase (*FADS1*) and the Δ6 desaturase (*FADS2*) enzymes. The *FADS* genes are in head-to-head orientation on chromosome 11, and common variation in the region is typically thought to be in nearly complete linkage disequilibrium [29,30,31]. This allows any of the commonly occurring SNPs in the *FADS* gene cluster to be used as a proxy for PUFA biosynthesis even if the lack of recombination sites in these genes have made it challenging to identify true causal variants in this region.

The *FADS* gene complex is composed of two major haplotypes: haplotype D (derived), which corresponds to major alleles, and haplotype A (ancestral), which corresponds to minor alleles. The distribution of the *FADS* haplotypes differs globally, with haplotype D more representative among persons with European (~62%) or African (~99%) ancestry, while haplotype A is more predominant among people native to the Americas (97%) [29]. Chilton et al. describe selection pressures for differing haplotype distributions in these various populations extensively [32]. Functional analyses of the haplotypes determined that haplotype D is more efficient than haplotype A in synthesis of both long-chain ω-3 and ω-6 PUFAs [29,33].

Haplotype A has been associated with higher plasma and tissue levels of LA and lower levels of AA, EPA, and DHA as well as reduced inflammation and CVD risk [34]. Secondary analyses of the VITAL ω-3 PUFA supplementation trial found that African Americans, who likely have haplotype D, have marked reductions in MI and CVD incidence over 5 years from ω-3 PUFA supplementation, compared with White and Hispanic participants, suggesting that while participants with either haplotype may have an ω-6/ω-3 imbalance, the increased efficiency and biosynthesis due to haplotype D can be maximized with the addition of dietary ω-3 PUFAs [32].

As others have performed, in this analysis, we use variant rs174547 (chr11:61,803,311; MAF = 32.4% in European samples) in *FADS*1, which has been previously studied in children, adults, and elderly, in various ethnic populations and diseases as a proxy for the *FADS* gene complex, with the minor allele (C) corresponding to haplotype A [23,26,35,36,37,38,39,40,41,42,43,44,45,46,47,48,49]. Previous explorations of this SNP tend to focus on the predictive capability of FA concentrations on inflammation [24,39], cognition [50], CVD [51,52], MetSyn [53], and lipid outcomes [44,54]. In this analysis, we hypothesize various FAs will act as mediators/suppressors and/or moderators of the association between *FADS* and lipid outcomes. As a mediator, we predict FAs not subject to *FADS* enzymes (like DHA) from the diet will act as indirect effectors on lipid outcomes. As a suppressor, we predict substrates of the *FADS* enzymes (like LA) will accentuate the higher (or lower) efficiency associated with each haplotype. Finally, as a moderator, we predict higher levels of substrates and lower levels of products will be associated with increased disparity in lipid outcomes between *FADS* groups.

## 2. Methods

### 2.1. UK Biobank Dataset

Use of participant data in this study was approved by the UKB (Project ID 85092), which consented all participants for the use of their medical and genetic data. This research was performed at the Pennsylvania State University (PSU) on university computers with data protection protocols. The PSU Institutional Review Board approved the use of the de-identified human subject data in this study.

The UKB recruited 500,000 volunteer participants between 40 and 69 years of age recruited from 2006 to 2010 in England, Scotland, and Wales. Details of the design of the study have been described [55]. Participants with demographic and lifestyle variables, lipid biomarkers, and NMR FA data were included and analyzed cross-sectionally. As NMR data were only collected on a subsample, we eliminated outliers across all included variables prior to eliminating participants with missing data. To trim the full dataset to our analytical subsample, first we eliminated outliers within each variable included in analysis; then, we removed participants with missing data in any of the variables. Our outlier handling included removing the top and bottom 0.25% of each outcome (*N* = 7222) and FA (*N* = 4741), with 451 participants overlap; so, we removed 11,512 participants as potential outliers and had 490,847 participants remaining. Next, we removed participants with missing covariate data (*N* = 3613), outcome data (*N* = 72,513), genetic data (*N* = 14,136), and FA data (*N* = 225,020) for a total of 260,988 participants removed. This left us with an *N* = 229,859 to complete our analysis.

### 2.2. Variables

EDTA plasma samples were collected at baseline recruitment by Nightingale (Nightingale Health Plc; biomarker quantification version 2020, http://nightingalehealth.com/atlas (accessed on 1 August 2022)). We used 5 measured FAs (or classes of FAs), specifically, the percentages of total ω-3 PUFAs, total ω-6 PUFAs, DHA, LA, and total SFA out of the total amount of FAs. Additionally, we used two calculated FA measures, non-DHA ω-3 PUFA (DHA subtracted from total ω-3 PUFA) and non-LA ω-6 PUFA (LA subtracted from total ω-6 PUFA), as predictor variables for a total of 7 FA measures. DHA and total ω-3 PUFAs were log-transformed to create a normal distribution in the analytical sample. Quartiles of FA measures were utilized to compare groups; the quartile table for each of 7 FA measures is summarized in Table S1. We considered four common lipid outcomes: TC (mg/dL), LDL-c (mg/dL), HDL-c (mg/dL), and TG (mg/dL) utilized in clinical practice. HDL-c and TG were log-transformed to create a more normal distribution in the analytical sample. We used exome sequence data in *FADS1* to classify the participant’s genotypes for rs174547, which were used as a proxy to determine a participant’s haplotype (D or A) [29]). *FADS* genotype was included in statistical analysis as a factor variable; *FADS* was coded by minor allele count of rs174547 as 0 = *FADS*-DD, 1 = *FADS*-DA, and 2 = *FADS*-AA. For the remainder of this manuscript, haplotype language (DD, DA, AA) will be used to simplify interpretation. Finally, we used age at time of FA measurement (years), sex (male and female), race/ethnicity (White, Mixed, Asian, Black), BMI (kg/m^2^), TDI, education level (college, associates, high school, no high school, unknown), smoking status (never, previous, current), alcohol use (never, previous, current), fish oil supplementation (yes or no), and cholesterol medication (yes or no) use as covariates in all analyses. The UKB participants self-reported race and/or ethnicity, which was subsequently categorized by UKB investigators into groups (White, Mixed, Asian, and Black), and as such, we will refer to this reported variable as race/ethnicity to encompass the variety of responses.

### 2.3. Statistical Analyses

Baseline characteristics of the total sample were evaluated between FADS groups using chi-square test for categorical variables and ANOVA for continuous variables.

**Figure 1:**
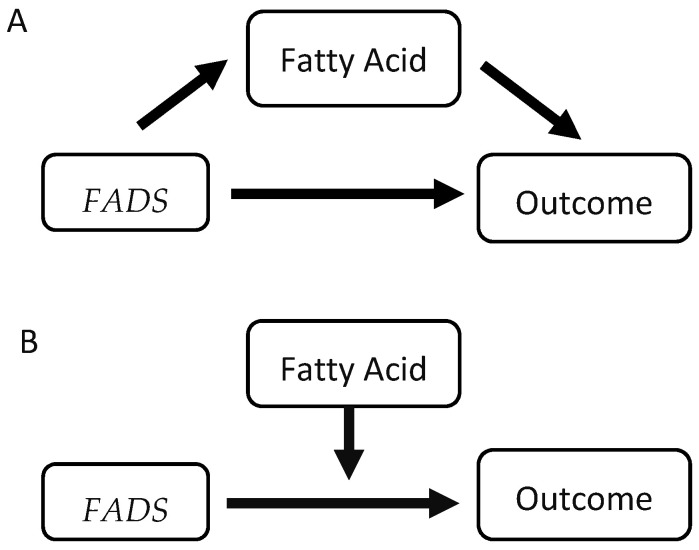
Framework of mediation and moderation Models. The direct association of the *FADS* with outcome, *FADS* with FA, and FA with outcome each must be statistically significant (*p* < 8.9 × 10^−4^) prior to analyzing any mediation (**A**) or moderation (**B**). Mediation was tested by comparing the FADS-only model (covariates + FADS) to the additive model (covariates + FA + FADS). Moderation was present if the interaction between the *FADS* and the FA was statistically significant (*p* < 8.9 × 10^−4^).

First, race/ethnicity was evaluated as a modifying factor of the *FADS*–lipid relationship and of the FA-dependent *FADS*–lipid relationship by including race in an interaction with *FADS* (race × *FADS*) and with *FADS* × FA (race × *FADS* × FA) and evaluating each against the null hypothesis with ANOVA (Figure 1). Next, we evaluated the potential mediating and/or moderating impacts of FAs on the *FADS*–lipids relationship using 56 linear regression models (7 FAs × 4 lipids × 2 hypotheses (mediation and moderation)) and following the standard approach of Baron and Kenney [56]. We fit separate linear models predicting each lipid by *FADS* after adjusting for 10 demographic and medical history covariates, estimating B_direct_ (the effect of the *FADS* genotype (0, 1, 2). The partial r^2^ of each model component was evaluated against the null hypothesis model (only covariates) with ANOVA. Following Baron and Kenney, we started by investigating evidence of moderation. The interaction term was added between *FADS* and the FA and tested against the additive model (FA + *FADS*) with ANOVA. For the mediation analysis, we separately added each FA to the model, estimating B_adjusted_ using an F-test to test whether the FA-adjusted model explained significantly (*p* < 8.9 × 10^−4^) more variation than the *FADS*-only model. We estimated the percent of the effect of *FADS* on the outcome that was mediated by the FA, as (B_direct_ − B_adjusted_)/B_direct_. We used a Bonferroni-adjusted significance threshold of *p* < 8.9 × 10^−4^ (*p* < 0.05/(2 models × 4 outcomes × 7 FAs) = 8.9 × 10^−4^) to determine statistical significance. *p*-values less than <2.0 × 10^−16^ are denoted as such R (Version 4.3.2, R Foundation for Statistical Computing.; Vienna, Austria) and JMP (Version 16.2, SAS Institute Inc.; Cary, NC, USA) were used for all analyses and GraphPad Prism (Version 9.4.1, GraphPad Software, Inc.; Boston, MA, USA) was used for figures.

## 3. Results

### 3.1. Sample Characteristics

The demographics of the UKB sample included in our analysis are summarized in Table 1. The cohort had a mean age of 57.1 years with more women (54%) than men and a mean BMI of 27.5 kg/m^2^. The sample was 95% White (British, Irish), while the remaining 5% was composed of participants who are Asian or Asian British (Indian, Pakistani, Bangladeshi, Chinese), Black or Black British (Caribbean, African), of Mixed ethnicity (White and Asian or Black), or who did not report race/ethnicity. The mean TDI was −1.4, meaning the sample, on average, was less deprived than typical for the UK. More than half of the participants were non-smokers (53.8%), most (92.1) reported current alcohol consumption, and 36.8% of the participants were college graduates with the majority (81.5%) having achieved a high school degree. Regular fish oil supplementation was reported in 31.4% of the sample.

### 3.2. Components of Mediation and Moderation Models

The first step in examining the impact of FAs on lipid outcomes was to determine bivariate associations including FADS–FA, FADS–FA, and FA–lipids (Figure 1). All three components of the models were statistically significant (*p* < 8.9 × 10^−4^), and the FADS–FA and FADS–FA are summarized in Table S2. The direct FADS–outcome effect is summarized in Table 2 along with evidence of mediation and moderation. The column “Evidence of Moderation”, lists the ANOVA *p*-value comparing the additive model (covariates + FA + FADS) to the interaction model (covariates + FADS × FA), and there is evidence of moderation in 19 models. The column “Evidence of Mediation”, lists the ANOVA *p*-value comparing the FADS-only model (covariates + FADS) to the additive model (covariates + FA + FADS), and there is evidence of mediation in 12 models and evidence of suppression in 16 models.

After confirming the direct associations between FA, *FADS*, and lipid outcomes, the race-/ethnicity-dependent association of *FADS* on each outcome was analyzed (Table S3). Participant race/ethnicity was a significant modifier of the association of *FADS* with TG (*p* = 1.9 × 10^−4^), as depicted in Figure S1; however, including race/ethnicity in a three-way interaction with FA and *FADS* determined that race/ethnicity of the participant was not a statistically significant (*p* < 8.9 × 10^−4^) modifier of the FA × *FADS* association with TG (Table S4). As there are no race-dependent differences in addition to FA-dependent differences, the analysis was conducted in the whole sample.

### 3.3. Moderation

**Figure 2:**
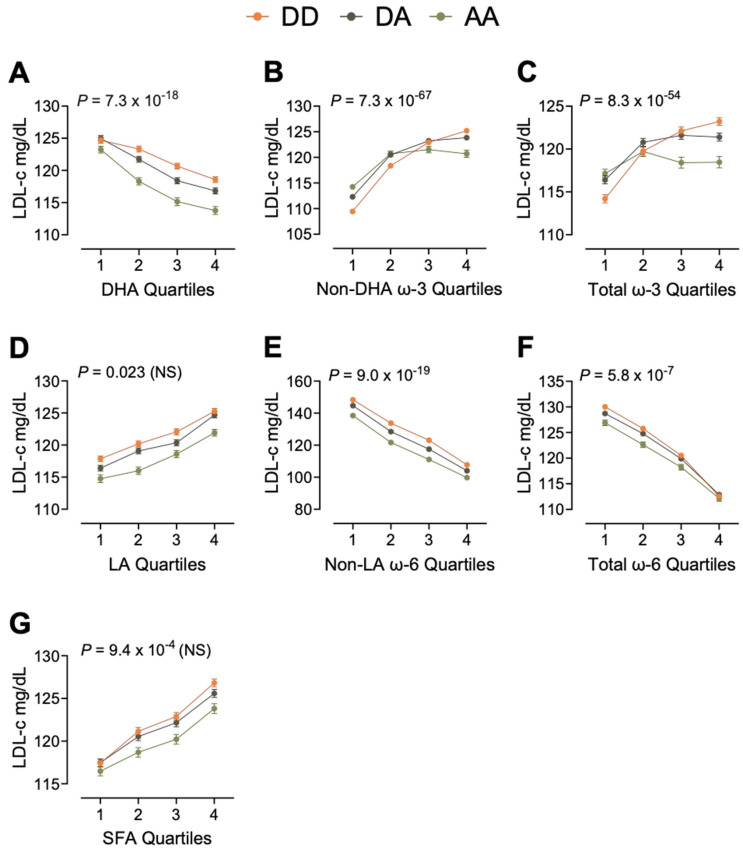
Moderation effect of each FA on the association between *FADS* and LDL-c (*N* = 229,859). The LSM and SEM for each group were plotted to depict the modifying effect of FA on the *FADS*–LDL-c association. Five of the seven FAs are statistically significant (*p* < 8.9 × 10^−4^) modifiers. (**A**) Plot of DHA quartiles predicting LDL-c. (**B**) Plot of non-DHA quartiles predicting LDL-c. (**C**) Plot of total ω-3 quartiles predicting LDL-c. (**D**) Plot of LA quartiles predicting LDL-c. (**E**) Plot of non-LA quartiles predicting LDL-c. (**F**) Plot of total ω-6 quartiles predicting LDL-c. (**G**) Plot of SFA quartiles predicting LDL-c.

For each of the 19 models showing evidence of moderation, Table 3 estimates the FADS–lipid relationship within each quartile of the FA. Briefly, non-DHA ω-3 PUFA, total ω-3 PUFAs, and non-LA ω-6 PUFA each moderated all the examined outcomes. LDL-c is described in detail to demonstrate the specific associations these FAs exert between the three genotype groups (Figure 2). The graphs of the remaining moderation models (TC, HDL-c, TG) are in Supplemental Figures S2–S4.

### 3.4. Example 1: Moderation of LDL-c

The effect of *FADS* on LDL-c was modified by five of the seven FA measures (Figure 2). While higher DHA was associated with lower LDL-c, with differences of roughly 5−10 mg/dL (Figure 2A), higher non-DHA and total ω-3 PUFA were associated with higher LDL-c, with the largest contrasts roughly 16 mg/dL for non-DHA ω-3 PUFAs and 9 mg/dL for ω-3 PUFAs (Figure 2B,C). For ω-6 PUFA, higher LA was associated with higher LDL-c, with differences of roughly 7 mg/dL (Figure 2D), while non-LA and total ω-6 PUFA were associated with lower LDL-c, with the largest contrasts roughly 40 mg/dL for non-LA ω-6 PUFAs and 16 mg/dL for ω-6 PUFAs (Figure 2E,F). Finally, SFA was associated with lower LDL-c, with differences of roughly 8 mg/dL (Figure 2G). Across each FA, the *FADS* minor allele was generally associated with lower LDL-c than common homozygotes (*FADS-*DD), however, stratified across each FA, differences between *FADS* groups were most evident with non-DHA ω-3 PUFAs and total ω-3 PUFAs. All comparisons were statistically significant (*p* < 8.9 × 10^−4^) unless otherwise stated.

There were minimal differences due to *FADS* at Q1 of DHA (Figure 2A) (*FADS*-DD vs. -DA: *p* = 0.44; *FADS*-DD vs. -AA: mean difference (95% CI) −1.6 (−2.8, −0.4)); however, at higher DHA, the minor allele was associated with lower LDL-c DHA, such that at Q4, *FADS*-DA had 1.6 mg/dL (−2.5, 0.8) lower LDL-c and *FADS*-AA had 4.6 mg/dL (−6.1, −3.1) lower LDL-c compared to *FADS*-DD. At Q1 of non-DHA ω-3 PUFA in Figure 2B, the minor allele is associated with higher LDL-c (DA: 2.8 mg/dL (1.9, 3.7); AA: 4.6 mg/dL (3.5, 5.7)); however, at higher levels of non-DHA ω-3 PUFA, these differences flip such that participants with the minor allele have lower LDL-c at Q4 (DA: −1.3 mg/dL (−2.2, −0.4); AA: −4.4 mg/dL (−6.2, −2.6)). Compared with *FADS*-DD, the overall difference between quartiles 1 and 4 was smaller in participants with one and two minor alleles (DD: −15.7 mg/dL; DA: 11.6 mg/dL; AA: −6.5 mg/dL).

Similar to non-DHA ω-3 PUFA, at Q1 of total ω-3 PUFA (Figure 2C), the minor allele was associated with higher LDL-c compared with *FADS*-DD (DA: 2.2 mg/dL (1.3, 3.2); AA: 2.9 mg/dL (1.7, 4.0)) and flips at higher levels of ω-3 PUFA. However, in this model, total ω-3 PUFA (Q4 vs. Q1) does not modify LDL-c in *FADS*-AA (*p* = 0.44), while *FADS*-DD and *FADS*-DA have higher LDL-c at Q4 compared with Q1 (DD: 9.0 mg/dL, DA: 5.0 mg/dL). LA does not modify *FADS* on LDL-c (Figure 2D). While there is statistical moderation between both *FADS* and non-LA ω-6 PUFA (Figure 2E), the differences between genotypes are similar across the quartiles. *FADS* groups also differ across levels of total ω-6 PUFAs (Figure 2F); while the minor allele is associated with lower LDL-c at Q1 of total ω-6 PUFA compared with *FADS*-DD (DA: −1.3 mg/dL (−2.0, −0.3), AA: −3.1 mg/dL (−4.4, −1.7)). There are no differences between groups at Q4 of ω-6 PUFA (DA: *p* = 0.26; AA: *p* = 0.18). SFA does not modify *FADS* on LDL-c (Figure 2G).

### 3.5. Mediation/Suppression

**Figure 3:**
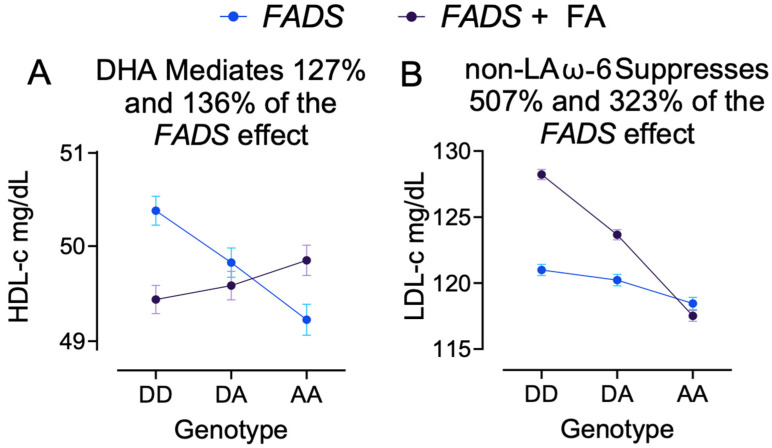
Examples of mediation and suppression. The figure depicts the graphical representation of both a mediation and suppression effect. In the mediation example, *FADS*-DA and -AA are associated with lower HDL-c; however, including DHA, the association of DA and AA is inversed compared to *FADS*-DD. In the suppression example, *FADS*-DA and -AA are associated with lower LDL-c, and including non-LA ω-6 PUFA reveals an even greater difference due to *FADS* suggesting that non-LA ω-6 PUFA is a suppressing factor of *FADS*. (**A**) Mediation plot of DHA mediating FADS association with HDL-c. (**B**) Mediation plot of non-LA suppressing FADS association with LDL-c.

Only nine models analyzed did not have evidence for moderation. Following Baron and Kenney, moderation is a more complicated, and therefore explanatory, model, so it is prioritized; however, mediation (or suppression) may occur with and without the presence of moderation and will be summarized below. Table 4 summarizes the estimated beta coefficients of FADS in the FADS-only model (covariates + FADS) and the additive model (covariates + FA + FADS), with FADS-DD as the reference. While 12 models suggest mediation (the indirect effect is stronger than the direct effect), there is evidence of suppression in the other 16 models (presence of a significant interaction) [57]. In these cases, inclusion of the FA strengthens the association of FADS with the outcome via both the beta and *p*-value. Visual examples of both mediation and suppression are provided in Figure 3.

### 3.6. Example 2: DHA Mediates FADS Effect on HDL-c

The impact of *FADS* on HDL-c is nearly completely mediated by DHA (Figure 3A). The association of participants with the minor allele with HDL-c is significantly different than the association of *FADS*-DD after adjusting for covariates (*p* < 2.0 × 10^−16^, adjusted difference (CI) logHDL: DD vs. DA −0.011 (−0.014, −0.008); *p* = 5.4 × 10^−32^); DD vs. AA −0.023 (−0.028, −0.018); *p* < 2.0 × 10^−16^. After including DHA in the model, the association of *FADS* with HDL-c is reduced (*p* = 1.1 × 10^−8^). The new adjusted associations of having one minor allele (DA vs. DD: 0.003 (0, 0.006); *p* = 1.2 × 10^−3^) and two minor alleles (AA vs. DD: 0.008 (0.004, 0.013); *p* = 3.9 × 10^−9^) reflect 127% and 136% mediation of *FADS* on HDL-c, respectively.

### 3.7. Example 3: Non-LA ω-6 PUFA Suppresses FADS Effect on LDL-c

While there were several potential models with suppression, we provide this example of non-LA ω-6 PUFA as a suppressor of *FADS* on LDL-c (Figure 3B). The association of the minor allele with LDL-c is significantly different than the association of *FADS*-DD after adjusting for demographic covariates (*p* < 2.0 × 10^−16^; DD vs. DA −0.7 (−1.2, −0.3); *p* = 1.2 × 10^−8^); DD vs. AA −2.5 (−3.2, −1.9); <2.0 × 10^−16^. After including non-LA ω-6 PUFA in the model, the association of the *FADS* with LDL-c remains highly significant (*p* < 2.0 × 10^−16^). The new adjusted associations of one minor allele (vs. DD: −4.5 (−4.9, −4.2); *p* < 2.0 × 10^−16^) and two minor alleles (vs. DD: −10.7 (−11.3, −10.1); *p* < 2.0 × 10^−16^) reflect 507% and 323% suppression of the association of the minor allele on LDL-c, respectively, prior to including non-LA ω-6 PUFA in the model.

## 4. Discussion

In this study, we demonstrate the validity and utility of a mediation and moderation framework to examine the inter-relationships between genetic variants, FAs, and lipid outcomes using the UKB. Where causal associations are known or proposed, mediation and moderation frameworks are useful to quantify the extent to which the association of the genotype is explained by the availability of the substrates or products of the *FADS* genes. Across the 56 tests conducted, we found evidence for mediation in 12, of suppression in 16, and of moderation in 19 models, suggesting FAs may be an explanation for the association of the *FADS* genes on outcomes. The value of these findings is evident in cases such as in the association of *FADS* with LDL-c, in which the rare haplotype is seemingly protected from higher LDL-c associated with higher ω-3 PUFAs, compared to *FADS*-DD and -DA.

We used the framework outlined in Figure 1 to show the roles FAs have in mediating *FADS*-dependent outcomes and verify that in some cases the mediating relationship is dependent on *FADS*. Prior work has characterized the association of each outcome with FA levels and with *FADS* SNPs, and we establish the magnitude of any causal relationship between them. Total SFAs are associated with higher TC [58,59], while LA and DHA are associated with lower TC, higher HDL-c, lower TG, and reduced risk for CVD [60,61,62,63] in interventional, cross-sectional, and case control studies. At the same time, the minor allele of *FADS* SNPs has been associated with lower TC, HDL-c, LDL-c, and TG in cross-sectional and interventional studies [44,64,65,66,67,68]. We provide evidence of the association of *FADS* on lipid outcomes depending on circulating FAs (moderation) and/or the association of *FADS* on lipid outcomes occurring via circulating FAs (mediation or suppression). In cases where moderation is present, such as non-DHA ω-3 PUFAs on LDL, the association of genotype with each outcome is fatty acid-dependent. Grundy et al. report percentage reduction in LDL-c as a reliable indicator of statin efficacy. Specifically, a lowering of LDL-c levels of 1% gives an approximate 1% reduction in the risk of atherosclerotic CVD, with more reduction at higher baseline levels of LDL-c [69]. Based on this, our findings suggest that *FADS*-DD participants with higher (Q4) total ω-3 PUFAs (Figure 2C) are at 7.9% greater risk for CVD events related to LDL-c compared to participants with the lowest total ω-3 PUFAs (Q1) (Table 5). In contrast, LDL-c is not modified by total ω-3 PUFAs in *FADS*-AA participants and so have no change in CVD risk.

Similar interactions to what we have proposed have been examined previously, using FA intake instead of circulating FA. Three studies, the Malmo Cancer and Diet study [44], the HELENA study [64], and the Doetinchem Cohort Study [66], each found that the minor allele of *FADS* SNPs was associated with lower TC and LDL-c; however, the interaction with FAs was not consistent. Minor alleles were associated with lower TC only at low-total ω-3 PUFA intake in the Malmo study, at high-ALA intake in the HELENA study, and at high-total ω-3 PUFA intake in the Doetinchem Study. While each of the studies provides insight into these gene–diet interactions, our analysis of circulating FAs provides further insight into the function of FAs in the body. Our results are most in line with the Doetinchem Study, specifically, we find that the minor allele (*FADS*-DA and -AA) is associated with lower TC, LDL-c, and HDL-c and higher TG; however, these associations independently depend on the level of several FAs. Minor alleles are associated with lower LDL-c at high non-DHA and total ω-3 PUFA; however, at low non-DHA and total ω-3 PUFA, major alleles are associated with lower LDL-c. The Doetinchem Study additionally examined ω-6 PUFA intake as a potential modifier, where, in the high-intake group, the minor allele was associated with lower HDL-c. In our analysis, we did not find total ω-6 PUFA to be a significant modifier of the association; however, LA and non-LA ω-6 PUFA were independent modifiers. Minor alleles were associated with lower HDL-c at all quartiles of LA (greater associations at Q2 and Q3) but only at high levels of non-LA ω-6 PUFA. Of note, we find that increasing non-LA ω-6 PUFA from Q1 to Q4, ~2.5% of total FAs, corresponds to an ~40 mg/dL lower LDL-c in all genotype groups. On top of this difference, participants with two minor alleles are ~10 mg/dL lower at all quartiles of non-LA ω-6 PUFA. The current standard prevention for participants at high risk for CVD is statins, which reduce LDL-c via HMG-CoA inhibition and upregulation of the LDL receptor to improve clearance. Statins can lower LDL-c between 20 and 50% and substantially reduce risk for developing or worsening CVD [69] however, based on our analyses, dietary modification of higher non-LA ω-6 PUFA may perform within the statin range by lowering LDL-c by ~25%. While LA comprises the majority of dietary ω-6 PUFA, differences in circulating LA had a smaller impact on the FADS–health relationship than non-LA ω-6 PUFA, which, while likely AA, could also be attributed to other ω-6 PUFAs.

After accounting for all moderation models, nine models remain that show evidence for mediation (or suppression) only. To our knowledge, there is no prior work to describe the potential mediating effect of FAs between *FADS* and lipid outcomes, so we will provide a statistical and biological interpretation of our results.

Mediation tests the validity of causal relationship between the independent, mediator, and dependent variables [70], in our case, the genetic variant, the FA measure, and the CVD biomarker outcome. This model considers the association of the FA, which confers the influence of an independent variable, genetic variation, onto an outcome, CVD biomarker. The results of our mediation analyses indicate the validity of FA-mediating genes and quantify the explanatory power of FAs in biological systems. For example, participants with the minor allele have lower HDL-c; however, this is completely explained by their correspondingly low DHA, which suggests these participants are targets for diet-dependent modifications of HDL-c by increasing DHA intake. Partial mediation was identified across the FAs and outcomes suggesting that FAs are not a complete explanation for genetic variation. However, they are likely a valuable and easily manipulated one that can modify several risk factors for CVD. Hence, for this example, our mediation findings can be stated as the association of *FADS* with differences in HDL-c occurs because of the impact *FADS* has on the level of DHA.

Suppression occurs when the direct association between the independent and dependent variables increases when the third variable is added to the model; the direct association is suppressed [28]. The inclusion of FAs in the model explains variability in association between *FADS* and the outcome, therefore, improving the explanatory power of *FADS*. In our analysis, we propose suppression can be described as the *FADS* enzyme requiring specific FAs to yield a specific outcome. For example, participants with the minor allele, hence, lower enzymatic efficiency, benefit from higher non-LA ω-6 PUFAs to have lower LDL-c. Without considering non-LA ω-6 PUFA, the association between the genotype and the outcome is unclear (or suppressed) due to potential negative feedback inhibition of the FA on the enzyme, but, within the context of non-LA ω-6 PUFAs, the association between *FADS* and outcome becomes more defined.

The focus on understanding FAs in the etiology of CVD, specifically how FA intake in the diet may contribute to prevention and treatment of the disease, is limited by lack of replication across previous work. While there is agreement regarding the potential benefits of ω-3 PUFA and potential drawbacks of SFA, disagreements regarding precise recommendations for prevention and treatment remain. Our approach addresses the gap two-fold: (1) using circulating FAs and continuous biomarkers to more precisely document effects and (2) including a more specific profile of FAs. General recommendations are not equipped to address the spectrum of health needs within a diverse population. Additionally, the variety of dietary recommendations around the world suggests that different populations adapt to their cultural intake and become accustomed to thrive. For example, the most prevalent fat in Europe varies by region: in northern Europe, the diet consists of mainly milk fat, whereas southern Europeans utilize olive oil predominantly [71]. The people historically from each region have adapted to benefit from the respective fat source; however, in the last 50 years, food patterns in southern Europe have increased meat and dairy consumption, which have increased risk for CVD [72]. Understanding individual differences in FA metabolism will elucidate how to maximize the potential benefits while minimizing the potential drawbacks.

Limitations: We used continuous markers of disease rather than hard diagnostic outcomes due to reliability and stronger causal inferences; however, we appreciate the value that diagnostic outcomes would have for clinical practice, and future studies should explore these relationships. We did not exclude participants on the basis of age or presence of disease, as this was a representative sample of the UKB cohort, not necessarily a healthy population. Additionally, the majority of our evidence suggests that *FADS* is relevant and descriptive in the White population within this sample, as it constitutes the majority (>95%) of participants; however, this may limit generalizability of results across populations. As there is no evidence of FA-dependent differences in the effect of *FADS* on lipid outcomes between racial/ethnic groups, we suggest that results found are reliable across groups; however, it is possible other enzymes and genes have more explanatory power in those populations with higher sample sizes. Given the cross-sectional nature of this study, future work should explore causality and lipid trajectories via temporal models.

In conclusion, this study has demonstrated the utility of examining the potential mediating and moderating effects of FAs in the human body on genetic associations with CV health markers. We find that several circulating FAs, including ω-3 PUFA, ω-6 PUFA, and SFAs, may be acting in the *FADS* enzymatic causal pathway in the UKB population. We also build on the previous evidence of *FADS* genotypes associated with CV health markers and show that different profiles of FAs affect the degree of that association with the *FADS* genotypes. Future functional studies within prospective cohorts and/or interventional studies are needed to better understand the potential causal and directional associations suggested by this analysis.

## Figures and Tables

**Table 1:** Baseline characteristics of total sample and split by *FADS* genotype (DD, DA, AA), (*N* = 229,859). Subset of FAs (DHA and total ω-3 PUFA) and outcomes (HDL-c and TG) are log-transformed.

Variables	Total Sample (*N* = 229,859)	*FADS*-DD Sample (*N* = 107,680)	*FADS*-DA Sample (*N* = 96,308)	*FADS-*AA Sample (*N* = 25,871)	*p*-Value ^1^
Women—*n* (%)	124,037 (54)	58,108 (54)	51,948 (53.9)	13,981 (54)	0.96
Age, years—Mean (SD)	57.1 (8.1)	57.0 (8.1)	57.2 (8.1)	57.3 (8.0)	2.2 × 10^−7^
Race/Ethnicity—*n* (%)					<2.0 × 10^−16^
White	218,314 (95.0)	99,735 (92.6)	93,549 (97.1)	25,030 (96.7)	
Mixed	1261 (0.5)	713 (0.7)	459 (0.5)	89 (0.3)	
Asian	4374 (1.9)	2742 (2.5)	1214 (1.3)	418 (1.6)	
Black	3057 (1.3)	2855 (2.7)	195 (0.2)	7 (0)	
Not Reported	2853 (1.2)	1635 (1.5)	891 (0.9)	327 (1.3)	
BMI, kg/m^2^—Mean (SD)	27.5 (4.8)	27.5 (4.8)	27.5 (4.8)	27.4 (4.7)	4.6 × 10^−4^
TDI—Mean (SD)	−1.4 (3.1)	−1.3 (3.1)	−1.5 (3)	−1.5 (3)	<2.0 × 10^−16^
Education—*n* (%)					1.7 × 10^−5^
College degree	84,542 (36.8)	39,664 (36.8)	35,268 (36.6)	9610 (37.1)	
Associates degree	25,180 (11)	11,642 (10.8)	10,607 (11)	2931 (11.3)	
High school degree	77,360 (33.7)	36,212 (33.6)	32,623 (33.9)	8525 (33)	
No high school degree	40,144 (17.5)	18,817 (17.5)	16,827 (17.5)	4500 (17.4)	
Unknown	2633 (1.1)	1345 (1.2)	983 (1)	305 (1.2)	
Smoking—*n* (%)					5.5 × 10^−6^
Never	125,108 (53.8)	59,046 (54.2)	51,995 (53.4)	14,067 (53.7)	
Previous	79,670 (34.3)	36,882 (33.8)	33,754 (34.7)	9034 (34.5)	
Current	24,000 (10.3)	11,181 (10.3)	10,148 (10.4)	2671 (10.2)	
Not Reported	1081 (0.5)	571 (0.5)	411 (0.4)	99 (0.4)	
Alcohol Intake—*n* (%)					<2.0 × 10^−16^
Never	9677 (4.2)	5098 (4.7)	3627 (3.8)	952 (3.7)	
Previous	7913 (3.4)	3793 (3.5)	3294 (3.4)	826 (3.2)	
Current	211,780 (92.1)	98,496 (91.5)	89,237 (92.7)	24,047 (92.9)	
Not Reported	489 (0.2)	293 (0.3)	150 (0.2)	46 (0.2)	
Fish Oil Supplement—*n* (%)	72,270 (31.4)	34,001 (31.6)	30,197 (31.4)	8072 (31.2)	0.38
Cholesterol Med Use—*n* (%)	40,357 (17.6)	19,099 (17.7)	16,777 (17.4)	4481 (17.3)	0.062
TC, mg/dL—Mean (SD)	220 (42.7)	220.6 (42.9)	220 (42.6)	217.3 (41.8)	<2.0 × 10^−16^
LDL-c, mg/dL—Mean (SD)	137.7 (32.6)	138 (32.8)	137.8 (32.6)	136 (32)	<2.0 × 10^−16^
HDL-c, mg/dL—Mean (SD)	56 (14.2)	56.3 (14.3)	55.8 (14.3)	55.2 (13.8)	<2.0 × 10^−16^
Total TG, mg/dL—Mean (SD)	152.6 (83.4)	149.7 (81.8)	154.5 (84.1)	157.4 (86.7)	<2.0 × 10^−16^
ω-3 PUFA, %—Mean (SD)	4.4 (1.5)	4.7 (1.4)	4.2 (1.4)	3.7 (1.5)	<2.0 × 10^−16^
DHA	2 (0.6)	2.1 (0.6)	1.9 (0.6)	1.8 (0.7)	<2.0 × 10^−16^
Non-DHA	2.4 (1)	2.6 (0.9)	2.3 (0.9)	1.9 (0.9)	<2.0 × 10^−16^
ω-6 PUFA, %—Mean (SD)	37.9 (3.4)	37.9 (3.5)	37.9 (3.4)	38 (3.4)	8.8 × 10^−8^
LA	28.9 (3.3)	28.6 (3.2)	29.1 (3.3)	29.8 (3.3)	<2.0 × 10^−16^
Non-LA	9 (1.9)	9.3 (1.9)	8.8 (1.8)	8.2 (1.7)	<2.0 × 10^−16^
Total SFA, %—Mean (SD)	34 (1.9)	34 (1.9)	34 (1.9)	33.9 (1.9)	<2.0 × 10^−16^

^1^ *p*-values calculated using chi-square test for categorical variables and ANOVA for continuous variables.

**Table 2:** Components of mediation and moderation models in whole sample (N = 229,859). The other two components of the models (*FADS*–FA and FA–Outcome) are each statistically significant (*p* < 8.9 × 10^−4^) in all models, and *p*-values can be found in Table S2. *FADS* is associated with each outcome. There are 12 models with evidence of moderation, 11 models with evidence of mediation, and 17 with evidence of suppression.

Outcome	FA	*FADS*-Only Model	Evidence of Moderation FA × *FADS*	Evidence of Mediation FA + *FADS*
		*p*-Value ^1^	*p*-Value ^2^	*p*-Value ^3^
TC	DHA	<2.0 × 10^−16^	6.1 × 10^−12^	<2.0 × 10^−16^
	Non-DHA ω-3		<2.0 × 10^−16^	3.73 × 10^−15^
	Total ω-3 PUFA		<2.0 × 10^−16^	3.3 × 10^−3^
	LA		0.014	<2.0 × 10^−16^
	Non-LA ω-6		4.0 × 10^−14^	<2.0 × 10^−16^
	Total ω-6 PUFA		0.034	<2.0 × 10^−16^
	Total SFA		2.1 × 10^−3^	<2.0 × 10^−16^
LDL-c	DHA	<2.0 × 10^−16^	<2.0 × 10^−16^	<2.0 × 10^−16^
	Non-DHA ω-3		<2.0 × 10^−16^	3.7 × 10^−13^
	Total ω-3 PUFA		<2.0 × 10^−16^	6.2 × 10^−3^
	LA		0.023	<2.0 × 10^−16^
	Non-LA ω-6		<2.0 × 10^−16^	<2.0 × 10^−16^
	Total ω-6 PUFA		5.8 × 10^−7^	<2.0 × 10^−16^
	Total SFA		9.4 × 10^−4^	<2.0 × 10^−16^
logHDL-c	DHA	<2.0 × 10^−16^	4.3 × 10^−6^	1.1 × 10^−8^
	Non-DHA ω-3		3.9 × 10^−15^	<2.0 × 10^−16^
	Total ω-3 PUFA		1.3 × 10^−8^	2.2 × 10^−10^
	LA		2.0 × 10^−4^	<2.0 × 10^−16^
	Non-LA ω-6		<2.0 × 10^−16^	1.3 × 10^−8^
	Total ω-6 PUFA		0.037	<2.0 × 10^−16^
	Total SFA		0.24	<2.0 × 10^−16^
logTG	DHA	<2.0 × 10^−16^	1.7 × 10^−13^	<2.0 × 10^−16^
	Non-DHA ω-3		<2.0 × 10^−16^	<2.0 × 10^−16^
	Total ω-3 PUFA		<2.0 × 10^−16^	<2.0 × 10^−16^
	LA		<2.0 × 10^−16^	<2.0 × 10^−16^
	Non-LA ω-6		<2.0 × 10^−16^	<2.0 × 10^−16^
	Total ω-6 PUFA		0.74	<2.0 × 10^−16^
	Total SFA		0.57	<2.0 × 10^−16^

^1^ *FADS*-only model *p*-values of ANOVA comparing null hypothesis (only covariates) and alternative model (outcome ~ *FADS*); ^2^ *p*-values of the ANOVA comparing additive model (outcome ~ FA + *FADS*) and interaction model (outcome ~ FA × *FADS*); and ^3^ *p*-values of the ANOVA comparing *FADS*-only model (outcome ~ *FADS*) and additive model (outcome ~ FA + *FADS*).

**Table 3:** Interquartile comparison in models with evidence of moderation (*N* = 229,859). Reference genotype DD for mean difference and 95% CI. Outcomes measured in mg/dL. Linear regression models fit *FADS* as a factor in an interaction with each FA. *FADS* was fit in a stratified analysis across each FA quartile to determine the impact of *FADS*-DA and -AA within each quartile.

Outcome	FA	*FADS* Group	FA Quartile 1 Mean Difference (95% CI)	FA Quartile 2 Mean Difference (95% CI)	FA Quartile 3 Mean Difference (95% CI)	FA Quartile 4 Mean Difference (95% CI)
TC	DHA	DD	−	−	−	−
		DA	−0.1 (−1.4, 1.1)	−2.3 (−3.4, −1.1)	−2.9 (−4, −1.8)	−2.2 (−3.3, −1.1)
		AA	−2.8 (−4.4, −1.2)	−7.1 (−8.9, −5.3)	−7.3 (−9.2, −5.3)	−6.5 (−8.5, −4.5)
	Non-DHA	DD	−	−	−	−
		DA	3.2 (2.1, 4.4)	2.9 (1.8, 4)	0.9 (−0.2, 2)	−1.4 (−2.5, −0.3)
		AA	5.2 (3.8, 6.7)	3.5 (1.7, 5.2)	−0.8 (−2.9, 1.3)	−5.7 (−8.1, −3.3)
	Total ω-3	DD	−	−	−	−
		DA	2.7 (1.5, 4)	1.6 (0.5, 2.7)	−0.5 (−1.7, 0.6)	−2.2 (−3.3, −1.1)
		AA	3.5 (2.1, 5)	0.5 (−1.4, 2.3)	−4.9 (−7, −2.8)	−6.3 (−8.5, −4)
	Non-LA	DD	−	−	−	−
		DA	−5.2 (−6.3, −4.1)	−6.7 (−7.7, −5.7)	−7.5 (−8.5, −6.6)	−5.7 (−6.6, −4.7)
		AA	−13.8 (−15.3, −12.3)	−16.3 (−17.8, −14.8)	−16.9 (−18.5, −15.2)	−12.5 (−14.4, −10.7)
LDL-c	DHA	DD	−	−	−	−
		DA	0.2 (−0.7, 1.1)	−1.6 (−2.5, −0.8)	−2.3 (−3.1, −1.4)	−1.6 (−2.5, −0.8)
		AA	−1.6 (−2.8, −0.4)	−5.1 (−6.5, −3.7)	−5.6 (−7.1, −4.1)	−4.6 (−6.1, −3.1)
	Non-DHA	DD	−	−	−	−
		DA	2.8 (1.9, 3.7)	2.2 (1.3, 3)	0.3 (−0.5, 1.2)	−1.3 (−2.2, −0.4)
		AA	4.6 (3.5, 5.7)	2.3 (1, 3.7)	−1.3 (−2.9, 0.3)	−4.4 (−6.2, −2.6)
	Total ω-3	DD	−	−	−	−
		DA	2.2 (1.3, 3.2)	1.1 (0.2, 1.9)	−0.5 (−1.4, 0.3)	−1.8 (−2.6, −0.9)
		AA	2.9 (1.7, 4.0)	0 (−1.4, 1.4)	−3.7 (−5.4, −2.1)	−4.7 (−6.5, −3)
	Non-LA	DD	−	−	−	−
		DA	−3.7 (−4.6, −2.8)	−5.2 (−6, −4.4)	−5.4 (−6.2, −4.7)	−3.7 (−4.4, −3)
		AA	−10 (−11.2, −8.9)	−12 (−13.2, −10.8)	−11.9 (−13.2, −10.6)	−8.2 (−9.5, −6.8)
	Total ω-6	DD	−	−	−	−
		DA	−1.1 (−2.0, −0.3)	−1 (−1.9, −0.2)	−0.5 (−1.4, 0.3)	0.3 (−0.5, 1.1)
		AA	−3.1 (−4.4, −1.7)	−3.2 (−4.5, −1.8)	−2.3 (−3.6, −0.9)	−0.5 (−1.7, 0.7)
logHDL-c	DHA	DD	−	−	−	−
		DA	0.005 (−0.001, 0.011)	0.009 (0.003, 0.015)	0.002 (−0.004, 0.008)	−0.003 (−0.009, 0.004)
		AA	0.013 (0.005, 0.021)	0.017 (0.007, 0.026)	0.01 (−0.001, 0.02)	−0.007 (−0.018, 0.004)
	Non-DHA	DD	−	−	−	−
		DA	−0.017 (−0.024, −0.01)	−0.019 (−0.025, −0.013)	−0.015 (−0.021, −0.009)	−0.004 (−0.011, 0.002)
		AA	−0.036 (−0.044, −0.028)	−0.042 (−0.051, −0.032)	−0.023 (−0.035, −0.012)	−0.005 (−0.018, 0.008)
	Total ω-3	DD	−	−	−	−
		DA	−0.012 (−0.019, −0.005)	−0.005 (−0.011, 0.001)	−0.003 (−0.009, 0.003)	0 (−0.006, 0.006)
		AA	−0.021 (−0.03, −0.013)	−0.005 (−0.015, 0.005)	−0.007 (−0.018, 0.005)	0.004 (−0.008, 0.016)
	LA	DD	−	−	−	−
		DA	−0.018 (−0.024, −0.012)	−0.021 (−0.028, −0.015)	−0.017 (−0.023, −0.011)	−0.013 (−0.019, −0.006)
		AA	−0.038 (−0.049, −0.027)	−0.047 (−0.058, −0.037)	−0.038 (−0.048, −0.029)	−0.028 (−0.037, −0.02)
	Non-LA	DD	−	−	−	−
		DA	0.008 (0.002, 0.015)	0.005 (−0.001, 0.012)	−0.009 (−0.015, −0.003)	−0.019 (−0.026, −0.013)
		AA	0.015 (0.007, 0.023)	0.001 (−0.008, 0.011)	−0.027 (−0.037, −0.016)	−0.04 (−0.052, −0.027)
logTG	DHA	DD	−	−	−	−
		DA	−0.029 (−0.043, −0.014)	−0.041 (−0.053, −0.029)	−0.023 (−0.035, −0.012)	−0.009 (−0.02, 0.002)
		AA	−0.063 (−0.082, −0.045)	−0.105 (−0.125, −0.085)	−0.078 (−0.099, −0.057)	−0.033 (−0.053, −0.012)
	Non-DHA	DD	−	−	−	−
		DA	0.1 (0.087, 0.113)	0.115 (0.102, 0.127)	0.088 (0.076, 0.101)	0.014 (0, 0.028)
		AA	0.211 (0.195, 0.227)	0.24 (0.22, 0.259)	0.137 (0.113, 0.161)	−0.009 (−0.038, 0.019)
	Total ω-3	DD	−	−	−	−
		DA	0.075 (0.06, 0.091)	0.059 (0.046, 0.073)	0.029 (0.016, 0.042)	−0.013 (−0.026, 0)
		AA	0.145 (0.126, 0.164)	0.089 (0.066, 0.112)	0.012 (−0.013, 0.037)	−0.052 (−0.078, −0.026)
	LA	DD	−	−	−	−
		DA	0.063 (0.049, 0.076)	0.073 (0.061, 0.086)	0.05 (0.038, 0.062)	0.033 (0.022, 0.044)
		AA	0.131 (0.107, 0.154)	0.148 (0.128, 0.169)	0.119 (0.101, 0.138)	0.062 (0.046, 0.077)
	Non-LA	DD	−	−	−	−
		DA	−0.062 (−0.073, −0.05)	−0.076 (−0.086, −0.066)	−0.061 (−0.071, −0.052)	−0.024 (−0.033, −0.014)
		AA	−0.135 (−0.151, −0.12)	−0.165 (−0.181, −0.15)	−0.127 (−0.144, −0.11)	−0.074 (−0.093, −0.054)

**Table 4:** Evidence of mediation. Model comparisons split by *FADS* groups with *FADS*-DD as reference.

Outcome	FA	*FADS-*DD vs. -DA				
		*FADS*-Only Model ^1^ Mean (CI)	*p*-Value ^2^	Additive Model ^3^ Mean (CI)	*p*-Value ^4^	% Mediation
TC	DHA	−1.3 (−1.9, −0.7)	5.5 × 10^−14^	−1.9 (−2.5, −1.4)	<2.0 × 10^−16^	−51%
	Non-DHA			1.2 (0.6, 1.8)	3.3 × 10^−12^	192%
	ω-3 PUFA			0.2 (−0.4, 0.7)	0.35	112%
	LA			−1.7 (−2.3, −1.2)	<2.0 × 10^−16^	−34%
	Non-LA			−6.3 (−6.8, −5.8)	<2.0 × 10^−16^	−392%
	ω-6 PUFA			−1.1 (−1.7, −0.6)	8.2 × 10^−12^	11%
	SFA			−1 (−1.6, −0.5)	1.5 × 10^−9^	21%
LDL-c	DHA	−0.7 (−1.2, −0.3)	1.2 × 10^−8^	−1.4 (−1.8, −1)	<2.0 × 10^−16^	−87%
	Non-DHA			0.8 (0.4, 1.3)	4.0×10^−10^	210%
	ω-3 PUFA			0.1 (−0.4, 0.5)	0.70	107%
	LA			−1.2 (−1.6, −0.8)	<2.0 × 10^−16^	−61%
	Non-LA			−4.5 (−4.9, −4.2)	<2.0 × 10^−16^	−507%
	ω-6 PUFA			−0.6 (−1.1, −0.2)	5.0 × 10^−7^	13%
	SFA			−0.6 (−1.1, −0.2)	1.9 × 10^−6^	17%
logHDL	DHA	−0.011 (−0.014, −0.008)	<2.0 × 10^−16^	0.003 (0, 0.006)	1.2 × 10^−3^	127%
	Non-DHA			−0.013 (−0.016, −0.01)	<2.0 × 10^−16^	−18%
	ω-3 PUFA			−0.004 (−0.008, −0.001)	2.3 × 10^−6^	59%
	LA			−0.017 (−0.02, −0.014)	<2.0 × 10^−16^	−58%
	Non-LA			−0.004 (−0.007, −0.001)	4.9 × 10^−6^	61%
	ω-6 PUFA			−0.012 (−0.015, −0.009)	<2.0 × 10^−16^	−7%
	SFA			−0.01 (−0.013, −0.007)	<2.0 × 10^−16^	10%
logTG	DHA	0.024 (0.017, 0.031)	<2.0 × 10^−16^	−0.025 (−0.031, −0.019)	<2.0 × 10^−16^	204%
	Non-DHA			0.075 (0.068, 0.081)	<2.0 × 10^−16^	−212%
	ω-3 PUFA			0.033 (0.026, 0.04)	<2.0 × 10^−16^	−37%
	LA			0.056 (0.05, 0.062)	<2.0 × 10^−16^	−134%
	Non-LA			−0.056 (−0.061, −0.051)	<2.0 × 10^−16^	334%
	ω-6 PUFA			0.03 (0.025, 0.034)	<2.0 × 10^−16^	−23%
	SFA			0.028 (0.022, 0.035)	<2.0 × 10^−16^	−19%
		**FADS-DD vs. -AA**				
TC	DHA	−4.2 (−5, −3.3)	<2.0 × 10^−16^	−5.6 (−6.5, −4.7)	<2.0 × 10^−16^	−35%
	Non-DHA			1.7 (0.8, 2.6)	2.6 × 10^−10^	141%
	ω-3 PUFA			−0.8 (−1.7, 0.2)	6.0 × 10^−3^	82%
	LA			−5.2 (−6, −4.3)	<2.0 × 10^−16^	−24%
	Non-LA			−15 (−15.8, −14.2)	<2.0 × 10^−16^	−261%
	ω-6 PUFA			−3.7 (−4.6, −2.8)	<2.0 × 10^−16^	11%
	SFA			−3.6 (−4.5, −2.7)	<2.0 × 10^−16^	14%
LDL-c	DHA	−2.5 (−3.2, −1.9)	<2.0 × 10^−16^	−4 (−4.7, −3.3)	<2.0 × 10^−16^	−57%
	Non-DHA			1.3 (0.6, 2)	1.0 × 10^−9^	150%
	ω-3 PUFA			−0.6 (−1.3, 0.1)	4.4 × 10^−3^	76%
	LA			−3.6 (−4.3, −2.9)	<2.0 × 10^−16^	−41%
	Non-LA			−10.7 (−11.3, −10.1)	<2.0 × 10^−16^	−323%
	ω-6 PUFA			−2.2 (−2.9, −1.5)	<2.0 × 10^−16^	13%
	SFA			−2.3 (−2.9, −1.6)	<2.0 × 10^−16^	11%
logHDL	DHA	−0.023 (−0.028, −0.018)	<2.0 × 10^−16^	0.008 (0.004, 0.013)	3.9 × 10^−9^	136%
	Non-DHA			−0.029 (−0.034, −0.024)	<2.0 × 10^−16^	−24%
	ω-3 PUFA			−0.009 (−0.014, −0.004)	1.7 × 10^−9^	61%
	LA			−0.037 (−0.042, −0.032)	<2.0 × 10^−16^	−60%
	Non-LA			−0.008 (−0.012, −0.003)	2.0 × 10^−7^	67%
	ω-6 PUFA			−0.025 (−0.03, −0.021)	<2.0 × 10^−16^	−10%
	SFA			−0.021 (−0.026, −0.016)	<2.0 × 10^−16^	10%
logTG	DHA	0.042 (0.031, 0.052)	<2.0 × 10^−16^	−0.068 (−0.078, −0.058)	<2.0 × 10^−16^	264%
	Non-DHA			0.163 (0.153, 0.173)	<2.0 × 10^−16^	−293%
	ω-3 PUFA			0.066 (0.055, 0.077)	<2.0 × 10^−16^	−59%
	LA			0.112 (0.102, 0.121)	<2.0 × 10^−16^	−169%
	Non-LA			−0.131 (−0.139, −0.123)	<2.0 × 10^−16^	416%
	ω-6 PUFA			0.059 (0.052, 0.066)	<2.0 × 10^−16^	−43%
	SFA			0.051 (0.042, 0.061)	<2.0 × 10^−16^	−24%

^1^ *FADS*-only model (covariates + *FADS*); ^2^ *p*-value of ANOVA comparing the null hypothesis model (covariates only) and the additive model; ^3^ additive model (covariates + FA + *FADS*); and ^4^ *p*-value of ANOVA comparing the *FADS*-only model and the additive model.

**Table 5:** Percent difference in LDL-c at each quartile of each FA. FA least square means for each quartile used to calculate percent difference from quartile 1 (reference) with LDL-c as outcome.

*FADS* Genotype	FA Quartile	% Difference in LDL-c from Quartile 1						
		DHA	Non-DHA	ω-3 PUFA	LA	Non-LA	ω-6 PUFA	SFA
DD	1	−	−	−	−	−	−	−
DD	2	−1.1%	8.1%	4.9%	2.0%	−9.8%	−3.3%	3.2%
DD	3	−3.2%	12.3%	6.9%	3.6%	−17.0%	−7.3%	4.7%
DD	4	−4.9%	14.4%	7.9%	6.3%	−27.3%	−13.4%	8.0%
DA	1	−	−	−	−	−	−	−
DA	2	−2.6%	7.3%	3.8%	2.3%	−11.2%	−3.0%	2.6%
DA	3	−5.2%	9.7%	4.5%	3.4%	−18.8%	−6.8%	4.0%
DA	4	−6.5%	10.3%	4.3%	7.1%	−28.1%	−12.3%	6.9%
AA	1	−	−	−	−	−	−	−
AA	2	−4.0%	5.6%	2.2%	1.1%	−12.1%	−3.4%	1.9%
AA	3	−6.6%	6.4%	1.1%	3.3%	−19.8%	−6.8%	3.2%
AA	4	−7.7%	5.7%	1.1%	6.2%	−28.0%	−11.6%	6.3%

## Data Availability

This research has been conducted using the UK Biobank Resource under Application Number 85092. Data are available from the UK Biobank (https://www.ukbiobank.ac.uk/ (accessed on 1 August 2022)) for researchers who meet the criteria and gain approvals to access the research database from the UK Biobank.

## Supplementary Materials

The following supporting information can be downloaded at: https://www.mdpi.com/article/10.3390/nu17061089/s1, Table S1. Quartile table of 7 FA measures. Summary of 7 plasma FAs measured by NMR in the UKB and considered in our analysis. Other FAs not analyzed include Percent PUFA and Percent MUFA. The mean, standard deviation, and interquartile ranges are provided for the N = 229,859 participants included in further analyses; Table S2. All models of FADS predicting FA and FA predicting outcome are significant. The other two components of the mediation and moderation; FADS predicting FA (FA ~ FADS) and FA predicting outcome (Outcome ~ FA) are each highly significant with most *p*-values < 1.0; Table S3. Sensitivity Test for Interactions with Race, N = 227,006. Excluded participants without race reported. *p*-values from ANOVA comparing the additive (FADS + race) to the interaction (FADS × race); Table S4. FA-dependent association of FADS with TG is not race-dependent. ANOVA *p*-values comparing null hypothesis (race×FA + race×FADS + FA×FADS) to the 3-way interactions model (race×FA×FADS) on TG. There are no significant interactions between race, FADS, and FAs. Figure S1. Race-dependent modification of FADS on TG; Figure S2. Moderation models predicting TC. Four of the 7 FA measures modify the FADS-effect on TC (N = 229,859). Results of least square mean estimates and SEM for the modifying effects of the 7 FA measures on FADS; Figure S3. Moderation models predicting HDL-c. Five of the 7 FA measures modify the FADS-effect on HDL-c (*N* = 229,859). Results of least square mean estimates and SEM for the modifying effects of the 7 FA measures on FADS; Figure S4. Moderation models predicting TG. Five of the 7 FA measures modify the FADS-effect on TG (*N* = 229,859). Results of least square mean estimates and SEM for the modifying effects of the 7 FA measures on FADS.

## Abbreviations

AA	arachidonic acid
ALA	alpha-linolenic acid
ANOVA	analysis of variance
BMI	body mass index
CI	confidence interval
CVD	cardiovascular disease
DHA	docosahexaenoic acid
DPA	docosapentaenoic acid
EDTA	ethylenediaminetetraacetic acid
EPA	eicosapentaenoic acid
FA	fatty acid
FADS	fatty acid desaturase
GISSI	Gruppo Italiano per lo Studio della Sopravvivenza nell’Infarto miocardico
HDL-c	high-density lipoprotein cholesterol
JELIS	Japan EPA Lipid Intervention Study
LA	linoleic acid
LD	linkage disequilibrium
LDL-c	low-density lipoprotein cholesterol
NMR	nuclear magnetic resonance
ω-3	omega−3
ω-6	omega−6
PSU	Pennsylvania State University
PUFA	polyunsaturated fatty acid
SFA	saturated fatty acid
SNP	single-nucleotide polymorphism
TC	total cholesterol
TDI	Townsend’s Deprivation Index
TG	total triglycerides
UKB	United Kingdom Biobank
