# Supplementary material for: The Effect of Fatty Acid Desaturase on Cardiovascular Lipid Biomarkers Depends on Circulating ω-3 and ω-6 Polyunsaturated Fatty Acids in the UK Biobank
Source: Nutrients. 2025 Mar 20;17(6):1089. doi: 10.3390/nu17061089 (PMC11945029; doi:10.3390/nu17061089)
Supplement: Supplementary file 1 [file nutrients-17-01089-s001.zip › nutrients-3514019-supplementary.pdf]

Supplementary Material

Tables S1-S4
Figure S1-S4

Table S1. Quartile table of 7 FA measures. Summary of 7 plasma FAs measured by NMR in the UKB and considered in our analysis. Other FAs not analyzed include Percent PUFA and Percent MUFA. The mean, standard deviation, and interquartile ranges are provided for the N = 229,859 participants included in further analyses.

FA	Min (%)	Q1 (%)	Median (%)	Q3 (%)	Max (%)
Total ω3-PUFA	1.2	3.4	4.2	5.2	11.3
DHA	0.5	1.6	1.9	2.3	4.9
Non-DHA	0	1.7	2.3	2.9	7.4
Total ω6-PUFA	25.3	35.8	38.4	40.4	46
LA	18.0	26.8	29.1	31.2	38.4
Non-LA	2.5	7.6	8.8	10.1	19.8
SFA	28.6	32.8	33.9	35.1	40.9

Table S2. All models of FADS predicting FA and FA predicting outcome are significant. The other two components of the mediation and moderation; FADS predicting FA (FA ~ FADS) and FA predicting outcome (Outcome ~ FA) are each highly significant with most p-values < 1.0.

		**Model Components**	
**Outcome**	**FA**	***FADS* – FA**	**FA – Outcome**
TC	DHA	$<2.2\times10^{-308}$	3.5×10^{-169}
	Non-DHA ω3	$<2.2\times10^{-308}$	$<2.2\times10^{-308}$
	Total ω3	$<2.2\times10^{-308}$	4.6×10^{-87}
	LA	$<2.2\times10^{-308}$	7.8×10^{-163}
	Non-LA ω6	$<2.2\times10^{-308}$	$<2.2\times10^{-308}$
	Total ω6	2.4×10^{-12}	$<2.2\times10^{-308}$
	SFA	8.7×10^{-43}	$<2.2\times10^{-308}$
LDL-c	DHA	$<2.2\times10^{-308}$	$<2.2\times10^{-308}$
	Non-DHA ω3	$<2.2\times10^{-308}$	$<2.2\times10^{-308}$
	Total ω3	$<2.2\times10^{-308}$	$<2.2\times10^{-308}$
	LA	$<2.2\times10^{-308}$	$<2.2\times10^{-308}$
	Non-LA ω6	$<2.2\times10^{-308}$	$<2.2\times10^{-308}$
	Total ω6	2.4×10^{-12}	$<2.2\times10^{-308}$
	SFA	8.7×10^{-43}	$<2.2\times10^{-308}$
HDL-c	DHA	$<2.2\times10^{-308}$	$<2.2\times10^{-308}$
	Non-DHA ω3	$<2.2\times10^{-308}$	6.6×10^{-63}
	Total ω3	$<2.2\times10^{-308}$	$<2.2\times10^{-308}$
	LA	$<2.2\times10^{-308}$	$<2.2\times10^{-308}$
	Non-LA ω6	$<2.2\times10^{-308}$	$<2.2\times10^{-308}$
	Total ω6	2.4×10^{-12}	$<2.2\times10^{-308}$
	SFA	8.7×10^{-43}	$<2.2\times10^{-308}$
TG	DHA	$<2.2\times10^{-308}$	$<2.2\times10^{-308}$
	Non-DHA ω3	$<2.2\times10^{-308}$	$<2.2\times10^{-308}$
	Total ω3	$<2.2\times10^{-308}$	2.3×10^{-259}
	LA	$<2.2\times10^{-308}$	$<2.2\times10^{-308}$
	Non-LA ω6	$<2.2\times10^{-308}$	$<2.2\times10^{-308}$
	Total ω6	2.4×10^{-12}	$<2.2\times10^{-308}$
	SFA	8.7×10^{-43}	$<2.2\times10^{-308}$

Table S3. Sensitivity Test for Interactions with Race, N = 227,006. Excluded participants without race reported. P-values from ANOVA comparing the additive (FADS + race) to the interaction (FADS × race)

Outcomes	*FADS* × race P-value
TC	0.39
LDL-c	0.63
HDL-c	8.6×10^{-3}
TG	1.9×10^{-4}

Table S4. FA-dependent association of FADS with TG is not race-dependent. ANOVA P-values comparing null hypothesis (race×FA + race×*FADS* + FA×*FADS*) to the 3-way interactions model (race×FA×FADS) on TG. There are no significant interactions between race, *FADS*, and FAs.

Outcome	FA	race × FA × *FADS* P-value
TG	DHA	0.60
	non-DHA	0.10
	ω3-PUFA	0.19
	LA	0.020
	non-LA	0.37
	ω6-PUFA	0.16
	SFA	0.72

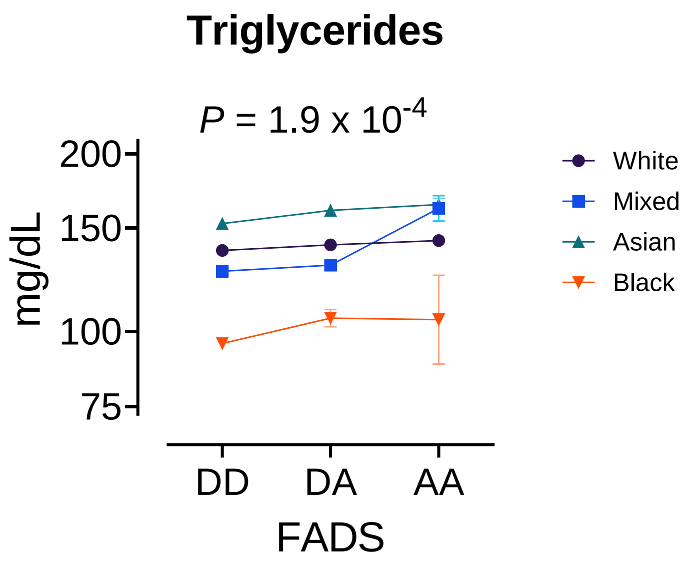

Figure S1. Race-dependent modification of FADS on TG.

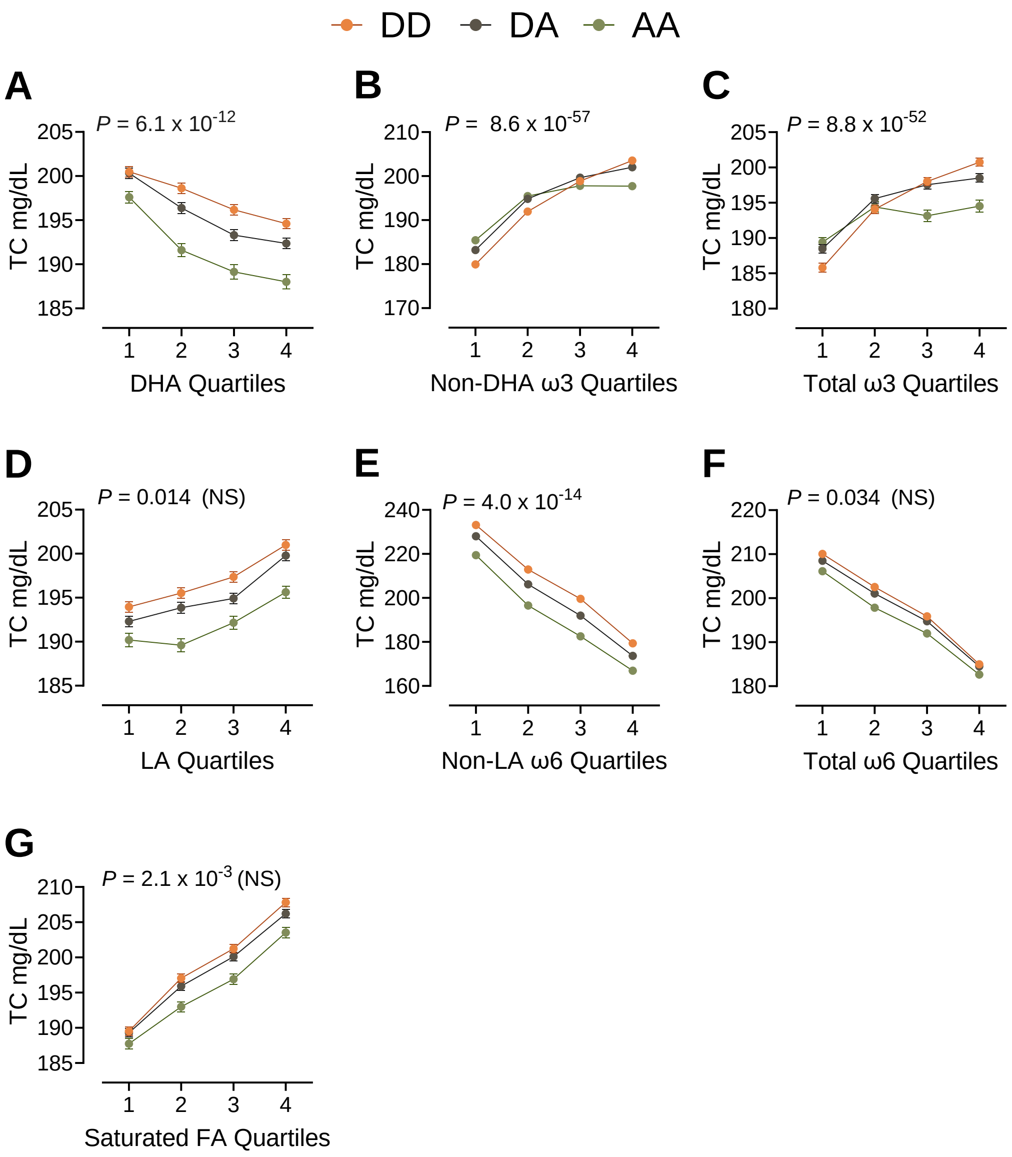

Figure S2. Moderation models predicting TC. Four of the 7 FA measures modify the FADS-effect on TC (N = 229,859). Results of least square mean estimates and SEM for the modifying effects of the 7 FA measures on FADS.

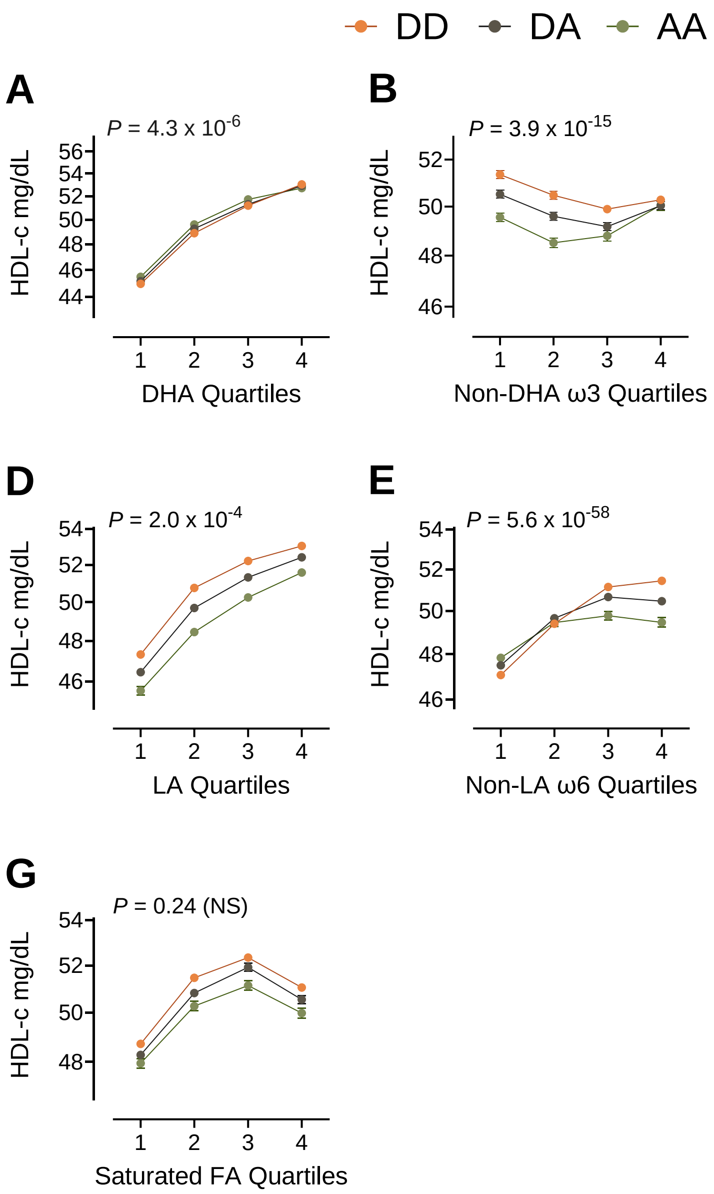

Figure S3. Moderation models predicting HDL-c. Five of the 7 FA measures modify the FADS-effect on HDL-c (N = 229,859). Results of least square mean estimates and SEM for the modifying effects of the 7 FA measures on FADS.

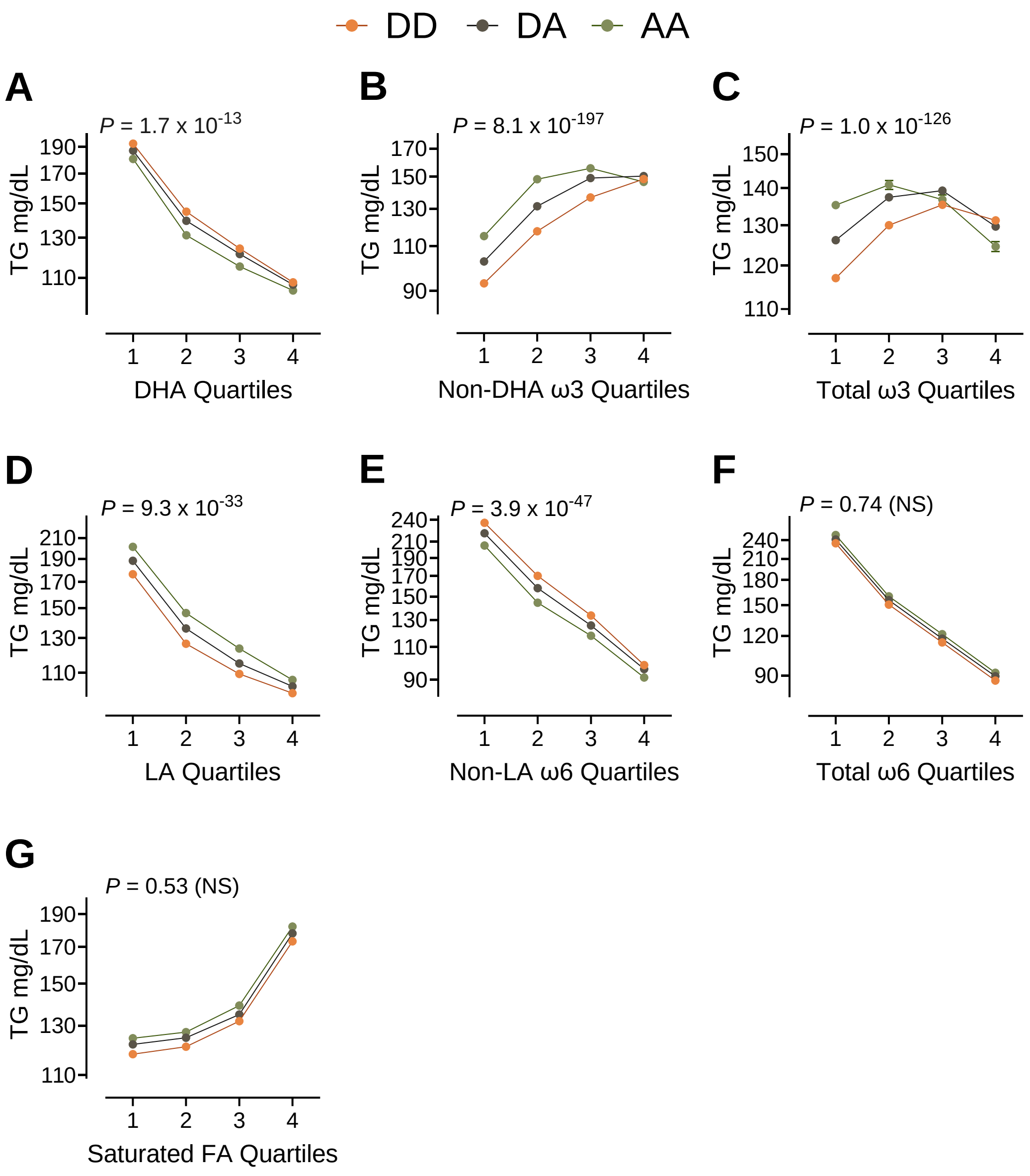

Figure S4. Moderation models predicting TG. Five of the 7 FA measures modify the FADS-effect on TG (N = 229,859). Results of least square mean estimates and SEM for the modifying effects of the 7 FA measures on FADS.
